# Deux espèces nouvelles de puces (Siphonaptera : Ctenophthalmidae & Rhopalopsyllidae) du Chili

**DOI:** 10.1051/parasite/2011183241

**Published:** 2011-08-15

**Authors:** J.-C. Beaucournu, L. Moreno, D. González-Acuña

**Affiliations:** 1 Laboratoire de Parasitologie médicale, Faculté de Médecine et Institut de Parasitologie de l’Ouest 2, avenue du Professeur Léon Bernard 35043 Rennes Cedex France; 2 Facultad de Ciencias Veterinarias, Universitad de Concepción casilla 537 Chillán Chile

**Keywords:** *Agastopsylla guzmani* n. sp., genre *Agastopsylla*, *Delostichus degus* n. sp., *Delostichus incisus*, *Delostichus ojedai*, Chili, *Agastopsylla guzmani* n. sp., genus *Agastopsylla*, *Delostichus degus* n. sp., *Delostichus incisus*, *Delostichus ojedai*, Chile

## Abstract

*Agastopsylla guzmani* n. sp. (Ctenophthalmidae) et *Delostichus degus* n. sp. (Rhopalopsyllidae) sont décrites. Une clé est donnée pour le genre *Agastopsylla ;* pour le genre *Delostichus*, une brève comparaison est faite avec *D. incisus* et *D. ojedoi* décrites depuis la parution du Catalogue de Smit en 1987.

## Abstract (en)

*Agastopsylla guzmani* n. sp. (Ctenophthalmidae) and *Delostichus degus* n. sp. (Rhopalopsyllidae) are described. A key is proposed for the genus *Agastopsylla*; for the genus *Delostichus*, a brief comparison is made with *D. incisus* and *D. ojedai* described after the issue of the Catalogue of Smit (1987).

## Abstract (es)

*Agastopsylla guzmani* n. sp. (Ctenophthalmidae) y *Delostichus degus* n. sp. (Rhopalopsyllidae) son descritos. Se propone una clave para el género *Agastopsylla* ; para el género *Delostichus*, se hace una breve comparación con *D. incisus* y *D. ojedai* descritas posterior a la publicación del catálogo de Smit (1987).

## Introduction

La richesse faunistique de la région chilio-andine ne cesse de s’affirmer et il est un peu frustrant, pour l’entomologiste, de constater que la totalité des Siphonaptères collectés le sont, non pas par des spécialistes de ce groupe, mais par des mammalogistes et/ou des ornithologistes amicaux ! Nous décrivons ici deux espèces nouvelles, l’une du nord du pays (Tarapacá) dans l’altiplano, l’autre du centre nord (Coquimbo).

## Résultats

### Famille Ctenophthalmidae Rothschild, 1915 Genre *Agastopsylla* Jordan & Rothschild, 1923 *Agastopsylla Guzmani* N. SP.

#### • Matériel de description

Mâle holotype sur *Akodon berlepschii*, Chungará (Tarapacá), coord. 18°15’ S – 69°09’ O, alt. 4 585 m, dans l’altiplano, 20 novembre 2007 (Jonathan Guzmán *rec*.). Pour Musser & Carleton (1993), ce rongeur est synonyme, ou sous-espèce, d’*Akodon albiventer* Thomas, 1897.

Dépôt du type dans les collections du premier auteur, ultérieurement déposé au Laboratoire d’Entomologie du Muséum National d’Histoire Naturelle de Paris.

*Derivatio nominis* : l’espèce est nommée en hommage à son collecteur, J. Guzmán, avec nos remerciements pour cette collecte, sans doute la plus haute, ou l’une des plus hautes du monde, pour un holotype !

#### • Description

Espèce évoquant *A. boxi boxi* Jordan & Rothschild, 1923 et *A. b. gibbosa* Beaucournu & Alcover 1990, par la présence sur la partie dorsale du tergite VII d’une touffe dense de soies de taille moyenne. *A. guzmani* n. sp. se sépare facilement de ces taxa par la structure du segment IX.

Capsule céphalique. OEil apparaissant rectangulaire, assez peu sclérifié. Palpe labial atteignant le trochanter ; palpe maxillaire un peu plus court que la coxa. Cténidie génale de quatre dents courtes, à apex arrondi, pratiquement incolores. Sétation céphalique apparemment comme chez *A. boxi* ssp. ; sétation occipitale, y compris le rang marginal de trois séries de soies : la 1^ère^ rangée avec une soie basale grande et cinq de longueurs décroissantes; la 2^ème^ rangée avec une grande et deux plus courtes ; la 3^ème^ avec la soie basale longue et quatre plus courtes.

Thorax : même chétotaxie que chez *A. boxi* ssp. ; cténidie prothoracique de 18 épines plus longues que la partie libre du segment. Mésothorax portant, d’avant en arrière, trois rangs de soies : respectivement 8 ou 9, 5 et 7. Métathorax portant une rangée principale de neuf longues soies. Métépimeron avec un spiracle petit, en “pomme de pin” ; neuf soies latérales. Tibia III montrant sept encoches tibiales et 12 soies latérales ; sa sétation est la suivante :

- la plus longue soie de ce tibia atteint l’apex du tarse I;
- la plus longue soie du tarse I (de la patte III) atteint l’apex du tarse II de cette même patte;
- la plus longue soie du tarse II (de la patte III) atteint l’apex du tarse IV de cette même patte (c’est l’apex du tarse III qui est atteint chez *A. boxi* ssp.);
- la plus longue soie du tarse III (de la patte III) atteint les 2/3 du tarse IV de cette même patte.

Le ratio des divers segments tarsaux est de l’ordre de 1 – 0,8 – 0,5 – 0,3 – 0,55.

Abdomen (segments non génitaux) : la rangée principale des tergites (du II au VII) comporte huit à neuf longues soies. Sur le VIIème, une touffe de soies de taille moyenne est située dorsalement ne dépassant qu’à peine la soie antépygidiale qui est en position relativement ventrale ; nous étudierons le tergite VIII plus loin. La chétotaxie des sternites (nous donnons entre parenthèses les chiffres correspondants chez *A. boxi* ssp.) est la suivante : st. II : 0 (0), 3 (2), 4 (2), 4 (2), 5 (2), 6 (3 ou 4).

**Figures 1-3.**
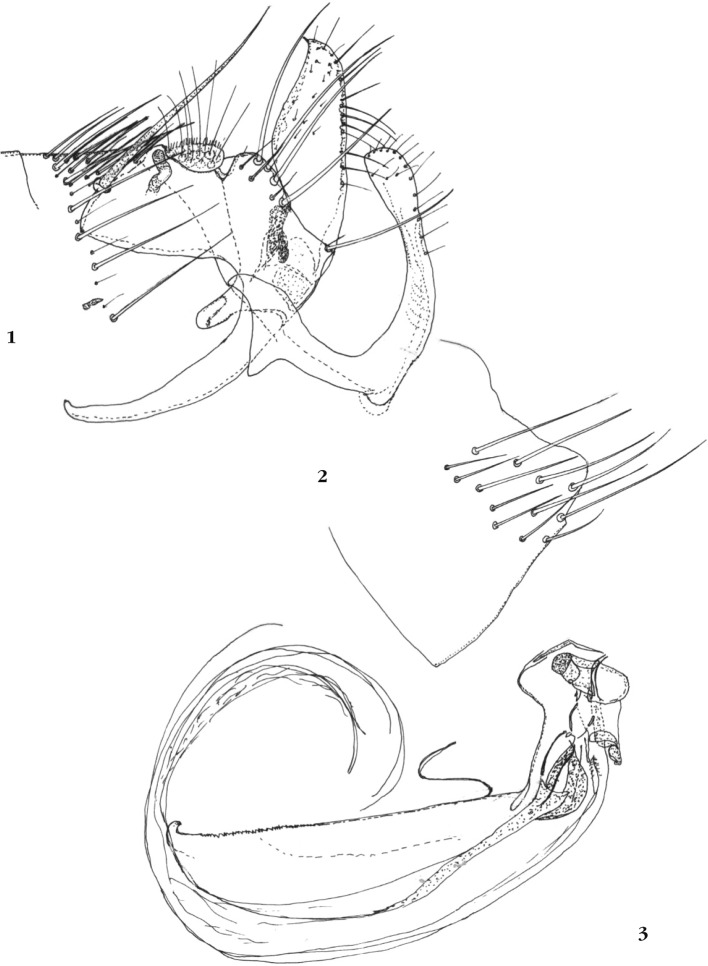
*Agastopsylla guzmani* n. sp., holotype. 1 : sternite VIII ; 2 : tergite VII, segment IX ; 3 : phallosome et endotendons.

Abdomen (segments génitaux) : le tergite VIII (figure 1) montre, le long de sa marge ventrale, un petit décrochement que l’on retrouve chez *A. boxi* ssp. ; ce segment porte dans sa moitié ventrale 13 soies plus ou moins longues; il y en a 20 ou plus chez *A. boxi* ssp. Segment IX (figure 2) : assez proche de celui des autres espèces pour ce qui concerne le basimère. En revanche, le télomère montre une convexité sur sa marge antérieure et un apex acuminé, antérograde. Chez *A. boxi* ssp. cet organe est au moins deux fois plus large et est, plus ou moins, quadrangulaire ; ce contour est assez proche de celui rencontré chez *A. pearsoni* Traub, 1952, *A. nylota nylota* Traub, 1952 ou *A. n. euneomys* Lewis, 1984. Le sternite IX, grêle et doucement arqué, évoque de nouveau cette fois *A. pearsoni* ou *A. nylota* ssp. Il est étroit au début du tiers distal et montre un apex doucement ovalaire, ne portant que des soies fines.

Phallosome (figure 3) : son bord apical est court par rapport à celui de *A. boxi* ssp. et diffère notablement de ceux des autre taxa, *A. nylota* ssp. en particulier. La marge dorsale est rétrograde, ce qui n’est pas le cas chez *A. boxi* ssp. *Hamulus* arrondi, *tubus* interior petit. *Lamina media* montrant une bordure en “rape” vers son apex. Tendons faisant une circonvolution.

Dimension de l’holotype : 1,4 mm.

#### • Discussion

La description de ce taxon nous a amené à le comparer aux autres espèces chez qui le mâle est connu. L’association de la touffe de soies sur le tergite VII et d’un sternite IX grêle, rend toute confusion impossible. En revanche, une nouvelle clé de détermination, faisant suite à celles de Johnson (1957), Hopkins & Rothschild (1966), Lewis & Spotorno (1984) et s’en inspirant *pro parte*, nous paraît utile.

### Clé actualisée du genre *Agastopsylla*

1. Mâles (ce sexe est inconnu chez *hirsutior*, mais la caractéristique de la femelle, n’étant pas un caractère sexuel secondaire, on étendra celle-ci au mâle) ..... 2
  - Femelles (ce sexe est inconnu chez *nylota* ssp. et chez *guzmani*) ............................................................ 8
2. Présence d’une touffe de soies sur le tergite VII, située dorsalement ; la soie antépygidiale est soit médiane par rapport à cette touffe, soit essentiellement postérieure ................................................................... 3
  - Pas de touffe de soies .............................................. 5
3. La soie antépygidiale est médiane par rapport à la touffe de soies ; le sternite IX est trapu .................... 4
  - La soie antépygidiale est située plus ou moins en arrière de la touffe de soies ; le sternite IX est grêle ......................................................................... *guzmani*
4. L’apex du sternite IX est modérément convexe et couvert dans sa partie dorsale de petites soies fines ......................................................................... *boxi boxi*
  - L’apex est fortement convexe et couvert dans sa partie dorsale de petites soies spiniformes … *boxi gibbosa*
5. Tibia III couvert, sur sa face externe, d’environ 22 soies (soies marginales exclues) ..................... *hirsutior*
  - Tibia III portant 15 ou moins de 15 soies sur sa face externe ......................................................................... 6
6. Télomère rectiligne, sa marge antérieure non recourbée, sa marge apicale seulement doucement oblique .............................................................. *pearsoni*
  - Télomère montrant un apex recourbé vers l’avant, sa marge fortement oblique ............................................ 7
7. Cténidie génale de quatre à cinq dents; palpe labial s’étendant jusqu’à l’apex de la coxa I; télomère plus que trois fois aussi long que large ......... *nylota nylota*
  - Cténidie génale de trois dents; palpe labial s’étendant au delà de l’apex de la coxa I ; télomère moins que trois fois aussi large que long .......... *nylota euneomys*
8. Tibia III couvert, sur sa face externe, d’environ 22 soies (soies marginales exclues) ..................... *hirsutior*
  - Tibia III portant 15 soies, ou moins de 15, sur sa face externe ......................................................................... 9
9. Conduit de la *bursa copulatrix* courbé brusquement vers l’avant près de sa terminaison .............. *boxi* ssp.
  - Conduit de la *bursa copulatrix* presque droit (légèrement courbé vers l’arrière près de sa terminaison) .......................................................................... *pearsoni*

### Famille Rhopalopsyllidae Genre *Delostichus* Jordan, 1942 *Delostichus Degus* n. sp.

#### • Matériel de description

Mâle holotype sur *Octodon degus* (Molina, 1782) n° 117, deux mâles et une femelle paratypes sur la même lame; deux mâles sur le même hôte, n° 104 ; un mâle, une femelle sur le même hôte, n° 106 ; quatre femelles sur le même hôte, n° 107 ; femelle allotype sur le même hôte, n° 108 ; un mâle, une femelle sur le même hôte, n° 116 ; un mâle paratype sur *Abrocoma bennettii* Waterhouse, 1837, n° 98 ; quatre mâles, une femelle sur le même hôte, n° 100; tous ces prélèvements effectués dans la Réserve Nationale “Las Chinchillas”, prov. du Choapa (Coquimbo), du 3 au 7 janvier 2010, coord. 31°28’ S – 71°03’ O, alt. 450 m (Lucila Moreno et Daniel González- Acuña *rec*.).

Une femelle isolée, de la même station, récoltée sur *Phyllotis darwini* (Waterhouse, 1837), n’appartient pas à ce taxon. Bien qu’ayant une spermathèque iden- tique, elle montre un *ductus bursae* sclérotisé, des soies courtes sur le scape, une chétotaxie du tergite VIII beaucoup plus pauvre. Il s’agit de *D. phyllotis* Johnson, 1957, décrite du Pérou sur *Phyllotis darwini*. Cette espèce est nouvelle pour le Chili.

Dépôt des types: mâle holotype, femelle allotype, quelques paratypes sont dans les collections du premier auteur, ultérieurement déposées au Laboratoire d’Entomologie du Muséum National d’Histoire Naturelle de Paris, les autres paratypes sont au Laboratorio de Zoología, Facultad de Ciencias Veterinarias, Universitad de Concepción, Avenida Vicente Méndez 595, Chillán, Chili.

*Derivatio nominis* : du nom spécifique de l’hôte apparemment préférentiel, *Octodon degus*, nom mis en apposition.

#### • Description

**Figures 4-9:**
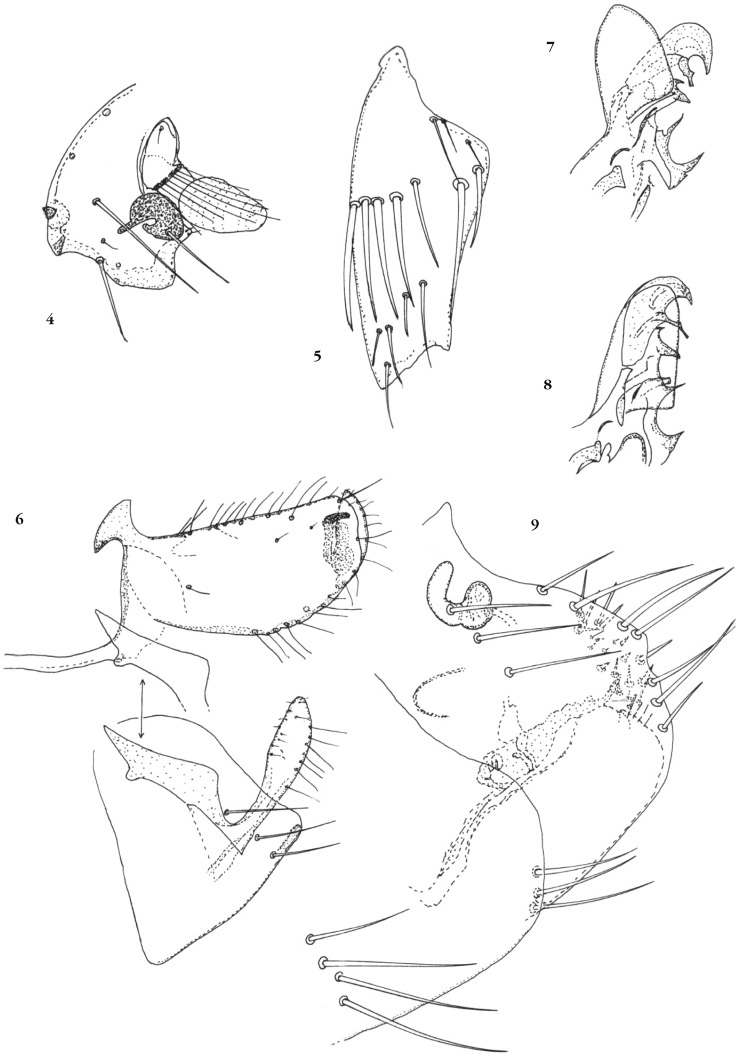
*Delostichus degus* n. sp. 4 : partie antérieure de la capsule céphalique del’allotype ; 5 : coxa I d’un mâle paratype; 6 : holotype, sternite VII et segment IX; 7 : holotype, apex du phallosome ; 8 : paratype, apex du phallosome ; 9 : allotype, segment VIII, sternite VII, spermathèque.

Capsule céphalique (figure 4) : deux soies préoculaires, une grande soie sous-oculaire accompagnée de deux très petites. Contour de la *gena* doucement concave. Soies du scape atteignant (lorsqu’elles sont parallèles à la massue) la moitié de celle-ci chez le mâle, l’apex pour les plus longues, chez la femelle. OEil très pigmenté montrant un lobe étroit antérograde et une invagination vers la moitié de son bord antérieur. Palpe labial atteignant, chez la plupart des spécimens, l’apex de la coxa I.

Thorax : coxa I (figure 5) montrant une rangée oblique de grosses soies et quelques autres de taille variable, situées surtout dans la partie inférieure. Le nombre des soies est très variable : la figure représente un mâle à chétotaxie particulièrement abondante. Coxa II montrant un *sulcus* interrompu dans son quart ventral.

Abdomen (segments non génitaux) : deux spinules sur le tergite I. Sternites : II avec deux à trois soies ; III et IV avec une soie ; V et VI avec deux ou trois soies.

Abdomen (segments génitaux du mâle) (figure 6) : sternite VIII montrant un repli cuticulaire à l’extrémité de son bord postéro-ventral, comme chez *D. talis*, par contre, la marge ventrale n’est pas encochée. Tergite IX : manubrium long, fin et doucement ondulé. Basimère beaucoup plus long que haut (comme chez *D. talis*), mais il n’est pas en continuité avec le manubrium (comme chez *D. smiti*) ; soies moins abondantes (*teste* Smit, 1987, fig. 51). Soie acétabulaire le plus souvent absente (elle a été ajoutée sur le dessin de l’holotype, car sa présence doit être la règle). Télomère un peu plus court que chez *D. talis*, de même longueur que chez *D. smiti*. Sternite : bras proximal fortement anguleux sur son bord postérieur ; bras distal fin à sa base, puis s’élargissant en ovale à apex arrondi. Toutes les soies sont fines, comme chez *D. smiti* qui montre une forme apicale plus large.

Phallosome (figures 7 et 8) de structure complexe avec cinq formations acérées saillantes sur le bord postérieur. Nous avons figuré “deux types” de cet organe, l’un représenté dans notre série par un seul exemplaire, l’holotype (et évoquant *D. smiti*), l’autre présente chez tous les autres mâles (et évoquant *D. talis*) : elles ne diffèrent en fait que par le déploiement, ou non, de la membrane préapicale.

Abdomen (segments génitaux de la femelle, spermathèque et *ducti*) (figure 9) : sternite VII portant trois soies et montrant un large lobe arrondi sur sa marge postérieure. Tergite VIII arrondi, avec un décrochement de la marge vers son milieu ; sept fortes soies marginales externes et trois ventrales ; nombreuses soies épaisses, quasi spiniformes, sur le tiers distal de la face interne : elles semblent courtes par un effet d’optique, étant toutes plus ou moins perpendiculaires à la cuticule de ce segment. Sclérifications unciformes sur la face latérale de ce segment, ce qui semble inédit dans ce genre. Sternite VIII grand, montrant les classiques petites soies distales.

Les *ducti* ne sont sclérotisés en aucun point chez cette espèce. Spermathèque évoquant celle de *D. octomyios* et, plus ou moins, celles de *D. smiti* ou de *D. talis*.

Dimensions : mâle, 1,1 à 1,4 mm (holotype : 1,3) ; femelle, 1,6 à 1,8 mm (allotype : 1,7).

#### • Discussion

*D. degus* n. sp. est manifestement proche de *D. talis* et de *D. smiti*. Il s’en sépare chez le mâle par la réunion chez cette espèce de deux caractères, connus l’un chez *talis*, l’autre chez *smiti* : repli cuticulaire du sternite VIII et partie apicale arrondie du sternite IX. C’est de *D. smiti* qu’il serait le plus proche, mais le palpe labial est plus long chez *D. degus*, le phallosome est différent et la femelle montre des sclérifications unciformes.

Depuis la parution du Catalogue de Smit (1987), où sont étudiés les Rhopalopsyllidae, trois espèces sont décrites dans ce genre : *D. incisus* Beaucournu & Torres-Mura, 1987 et *D. ojedoi* Beaucournu & Gallardo, 2005, décrites d’Argentine et la présente espèce, du Chili. Les mâles de *D. incisus* et de *D. ojedai* ont un palpe labial plus court que la coxa I; le sternite VIII est acuminé à son apex postéro-ventral qui porte une touffe de longues soies chez *D. ojedai;* chez *D. incisus*, il montre un petit repli cuticulaire à cet endroit comme chez *D. talis* et *D. degus* n. sp., mais il est moins prononcé. Chez *D. ojedai*, l’apex du sternite IX est acuminé et est recouvert de soies fines; cet apex est moins acuminé chez *D. incisus* qui ne montre pas de soie sur sa marge dorsale. La femelle de *D. incisus* est immédiatement caractérisée à l’intérieur du genre *Delostichus* par une fine encoche sur la marge distale du sternite VII; celle de *D. ojedai* est inconnue.
